# Phylogenetic signal in tooth wear? A question that can be answered—By testing

**DOI:** 10.1002/ece3.5214

**Published:** 2019-05-09

**Authors:** Marcus Clauss

**Affiliations:** ^1^ Clinic for Zoo Animals, Exotic Pets and Wildlife, Vetsuisse Faculty University of Zurich Zurich Switzerland

## Abstract

Do all teeth show the same wear traces when processing the same diet, or do the wear traces of the same diet differ between species, maybe due to differences in tooth morphology or chewing physiology? Questions like this one can be tested using appropriate biological and statistical methods. Without such tests, claiming that a certain proxy of tooth wear represents a “taxon‐free” signal remains a hypothesis.

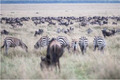

In a recent study, Fraser, Haupt, and Barr (2018) demonstrated that not only diet, but also tooth wear data show a phylogenetic signal. In the abstract, the authors state that “We suggest that morphological traits inherited from ancestral clades (e.g., tooth shape) *influence the ways in which the teeth wear during mastication* and constrain the foods individuals of a species can effectively exploit [my emphasis].” This interpretation not only suggests that phylogeny‐driven tooth shape influences the food that can be exploited—which would not be the real bone of contention about whether phylogeny needs to be accounted for in using tooth wear. It additionally hints, in the bolded part, at the possibility that some phylogeny‐related traits influence the way a food leaves wear traces on teeth. And this is the real bone of contention: that the same food might lead to different wear patterns in different species.

However, Fraser et al. (2018) did not test this—in order to do so, they would have had to test diet data versus wear proxies with and without accounting for phylogeny, recording whether there was phylogenetic signal in the association between the two. Such a test was actually published, in a less systematic way and without using statistics that formally account for phylogeny, by Mihlbachler, Campbell, Ayoub, Chen, and Ghani (2016), who showed that ruminants and perissodactyls, considered similar in the diet they ingest, did not show identical tooth wear patterns.

As a response to Fraser et al. (2018), DeSantis et al. (2018) dwell on the former implication—that phylogeny separates species of different diet niches, and that therefore the diet = tooth wear measure will necessarily contain a phylogenetic component. “Controlling” for this phylogenetic component, they argue, therefore obliterates the ecological diet signal. DeSantis et al. (2018) use the example of a hypothetical mammal community that consists of only two kinds of taxa—one taxon being herbivores and the other being carnivores, with classical differences in dental morphology as only the carnivores have a carnassial—and explain why in such a dichotomic situation, correcting for phylogeny, when correlating dental morphology and diet, will lead to a nonsignificant result. This example is similar to the classic introduction to phylogenetic statistics by Garland, Dickerman, Janis, and Jones (1993) with the surprising result that when controlling for phylogeny, home range sizes between carnivores and ungulate herbivores do not differ statistically (whereas in reality, in terms or square meters per body size, they do), or to the fact that because baleen filter feeding evolved in only one cetacean lineage, there is a clear negative relationship between body size and prey size in marine mammals in normal statistics, but not when controlling for phylogeny (Carbone, Codron, Scofield, Clauss, & Bielby, 2014). All these examples have in common that an ecological dichotomy (tooth morphology, home range size, and prey size) parallels a phylogenetic dichotomy (Carnivora vs. Ungulata, marine mammals vs. Mysticeti). The comparison of “conventional” statistics (significant in such cases) and phylogenetic statistics (nonsignificant in such cases) is relevant, because it reveals the phylogenetic structure of the data. The result could only be considered “noninstructive” if “nonsignificant” in one of the analyses was by default equated to “biologically irrelevant,” but we all know that we have to keep statistical significance and biological relevance apart when interpreting data. A “nonsignificant” result can have a very relevant meaning. If, in these three example datasets, a carnivorous and a herbivorous lifestyle with different tooth morphology or home range sizes, or filter feeding, would not only have evolved at a single crucial node of the underlying phylogeny, but if the data would include various lineages in which carnivores and herbivores, or filter feeding, had evolved in ecological convergence, then testing the corresponding dataset with phylogenetic statistics would not only be just as relevant, but would have the power to reveal whether the one ecological convergence (trophic level and filter feeding) is paralleled by another morphological or ecological convergence (tooth morphology, home range size, and prey size), or whether in spite of convergence in the one, there is no convergence in the other. Whether “nonsignificance” indicates “biological irrelevance” depends on the question asked, the statistical method, the sample composition, and the sample size. Many datasets are not composed of, and do not answer questions based on, simple dichotomies, but of more complex data structures, such as browsing and grazing feeding types that occur in various ungulate, rodent, and macropod lineages.

With respect to tooth wear, DeSantis et al. (2018) do not critically reflect on the implication that the same diet might cause different wear in different species. They understand tooth wear as a “function‐driven method,” and equate it with an x‐ray machine that will always detect knives carried by a virtual family at an airport security check. What they overlook in this example is that, to stay in the metaphor, phylogeny, or species‐specificity, could (but would not necessarily have to) mean that some families carry knives openly, some carry them inside of leather sheaths, some carry several knives in the same sheath, some carry lots of other radio‐dense litter in the same luggage, some wrap their knives in tin foil, and some carry knives with ceramic blades. And hence, depending on the phylogeny of the family, the detection signal of the x‐ray machine may vary. The real question that DeSantis et al. (2018) would have to answer is: in their hypothetical scenario of only two types of animals—herbivores and carnivores—would they predict that a certain diet—such as grass, or bone, or nuts—would lead to identical wear patterns in both groups? If you feed grass to a dog (and the dog eats it for a few days), would the microwear resemble that of a cow eating grass? Does the mesowear of a panda resemble that of a bamboo lemur or a sika deer? Or would the wear pattern differ because of differences in other traits (captured by a phylogenetic signal) such as dental morphology or chewing physiology? Only if one proved that a diet will always lead to the same dental wear irrespective of the species investigated, could the stance that phylogeny need not be accounted for when dealing with tooth wear proxies be defended. Maybe there are some dental morphology or wear proxies that have this characteristic, but that should then be demonstrated by testing. Recent findings on differences between phylogenetic groups (Mihlbachler et al., 2016) make this stance appear difficult to defend as a default option. Actually, even the classic publication that introduced mesowear (Fortelius & Solounias, 2000) already identified taxa (hyraxes, tragulids, and duikers) that appear to differ from the overall pattern. And the findings of Kaiser et al. (2013) that the correlation coefficient between diet and hypsodonty differs depending on whether phylogeny is accounted for or not, whereas that of mesowear did not change when phylogeny was accounted for, suggests that there are differences in this respect between different dental and wear proxies (in the phylogenetic sample of that dataset). This should be investigated, and not a priori claimed to matter, or not matter. Including results of both statistical approaches in comparative studies that use dental morphology or tooth wear proxies in relation to other variables, checking for the phylogenetic signal, and interpreting putative differences between the two statistical methods appears a prudent, diligent approach.
